# Epidemiology of *Clostridium difficile* infection in Asia

**DOI:** 10.1186/2047-2994-2-21

**Published:** 2013-07-01

**Authors:** Deirdre A Collins, Peter M Hawkey, Thomas V Riley

**Affiliations:** 1 Microbiology and Immunology, School of Pathology and Laboratory Medicine, the University of Western Australia, Perth, Australia; 2 West Midlands Public Health Laboratory, Health Protection Agency, Heart of England NHS Foundation Trust, Birmingham, UK; 3 School of Immunity and Infection, University of Birmingham, Birmingham, UK; 4 Division of Microbiology and Infectious Diseases, PathWest Laboratory Medicine (WA), Perth, Australia

**Keywords:** *Clostridium Difficile*, Clostridium Infections In Humans, Epidemiology

## Abstract

While *Clostridium difficile* infection (CDI) has come to prominence as major epidemics have occurred in North America and Europe over the recent decade, awareness and surveillance of CDI in Asia have remained poor. Limited studies performed throughout Asia indicate that CDI is also a significant nosocomial pathogen in this region, but the true prevalence of CDI remains unknown. A lack of regulated antibiotic use in many Asian countries suggests that the prevalence of CDI may be comparatively high. Molecular studies indicate that ribotypes 027 and 078, which have caused significant outbreaks in other regions of the world, are rare in Asia. However, variant toxin A-negative/toxin B-positive strains of ribotype 017 have caused epidemics across several Asian countries. Ribotype smz/018 has caused widespread disease across Japan over the last decade and more recently emerged in Korea. This review summarises current knowledge on CDI in Asian countries.

## Introduction

*Clostridium difficile* causes infection ranging from mild diarrhoea to pseudomembranous colitis (PMC), primarily in older age patients who have been exposed to antibiotics. Epidemics of *C. difficile* infection (CDI) have occurred in North America and Europe over recent decades and the epidemiology of CDI in these regions is well-documented. These epidemics have demonstrated the need for surveillance of the international movement of *C. difficile* strains [1]. Circulating strains in Asia, as in other regions, have the potential to spread internationally, warranting close monitoring of the prevalence and molecular epidemiology of CDI in the region. Indeed, it is likely that the variant toxin A-negative/ toxin B-positive (A^-^B^+^) ribotype 017 *C. difficile* strain originated in Asia. One particular clindamycin-resistant ribotype 017 strain of apparent clonal origin has dominated international typing studies of A^-^B^+^ strains and has been the cause of epidemics in Canada, the Netherlands and Ireland [2-4]. Unfortunately, limited data are available on CDI in Asia. A recent survey found that awareness of CDI in physicians is poor in Asia, with underestimation of its contribution to antibiotic-associated disease and recurrence rates [5]. In addition, comprehensive culture and toxin testing for *C. difficile* are lacking in many Asian hospitals. As a consequence, reports of *C. difficile* are rare in Asia, so data on prevalence and circulating strains are limited. What reports are available on Asian countries are described here.

### Literature search and selection strategy

PubMed searches were performed for publications prior to 1 May 2013 with the term “*Clostridium difficile*” combined with specific Asian country names. A search for “*Clostridium difficile*” was also performed on the Wanfang and KoreaMed databases. Manual searches of cited references of these articles were performed and relevant English language articles and abstracts were included for analysis.

### East Asia

#### Japan

No English language reports were found on the prevalence of CDI in Japan. However, molecular typing studies have provided some epidemiological information since the 1990s. A variety of typing techniques have been employed in Japan, including *tcdA* and *tcdB* characterisation [6,7], pulsed field gel electrophoresis (PFGE) [8], polymerase chain reaction (PCR) ribotyping [8,9] and *slpA* typing [10]. Application of molecular typing techniques has identified A^-^B^+^ strains in regions across Japan [6,11-14]. PCR typing of *tcdA* on six A^-^B^+^ strains from Japan and Indonesia identified indistinguishable repeating sequences with two deletions (1,548 and 273 nucleotides in size) [7]. A later study also grouped A^-^B^+^ strains from Japan and Indonesia, as toxinotype VIII and ribotype fr/017, with several subtypes identified by PFGE [15].

Several ribotyping studies have been performed, indicating predominance of ribotype “smz” over the past decade [8,16,17]. A 5-year study in a Tokyo hospital followed the proliferation of ribotype “smz”, peaking in 2004 (64% of cases) [16]. Ribotype “smz” is recognised internationally as ribotype 018 (personal observation), and three major subtypes have been identified within Japan by *slpA* typing, two of which are widespread [10,18]. Ribotype 018 strains have caused CDI recently in Korea [19], Austria, Spain and Slovenia [20], and have been responsible for outbreaks of disease in Italy since 2007 [21]. Other common ribotypes were 014, 002 and 001 [14,16,17]. Ribotype 027, which has caused widespread epidemics in North America and Europe, has been reported only occasionally in Japan [16].

#### Korea

A country-wide survey of 17 tertiary hospitals in Korea from 2004 to 2008 found that the incidence of CDI increased from 1.7/1,000 adult admissions to 2.7/1,000 admissions [22]. Diagnostic methods were not reported, and may have differed between sites or over time, contributing to these apparent increases.

Risk factors for recurrent CDI included antibiotic therapy, anaemia, and tube feeding in one study [23], while another found proton pump inhibitor (PPI) use alone was associated with recurrent disease [24]. One study found that the proportion of community-acquired CDI (CA-CDI) among all CDI cases within a Busan hospital was 7.1% [25], while another reported that 59.4% of cases of CDI presenting at the emergency department of a Seoul hospital were community-acquired [26].

Variant A^-^B^+^ strains have been common in Korea since the 1990s [27] and increased in prevalence among all strains significantly between 2002 and 2005, peaking at 50% in 2004 [27-30]. These epidemic A^-^B^+^ strains belonged to ribotype group 017 and were widespread in Korea [27,28,31]. Subtyping of 017 strains revealed six different PFGE pulsotypes and 13 restriction fragment length polymorphism-based subtypes [32]. Shin *et al*. reported that 72% of PMC cases between 2006 and 2010 were caused by A^-^B^+^ strains [29]. Ribotype 018 was the most prevalent strain isolated in Seoul from September 2008 to January 2010 [33]. Ribotype 027 was detected in 2006 [34] in a hospital-associated case of PMC but has failed to proliferate in Korea [19]. Type 078 was reported as the most common (3.1%) binary toxin-positive strain [31].

#### China

The lack of reports on CDI from mainland China contrasts with the relatively large number of reports from neighbouring Korea and Japan. An English language review of the Chinese language literature found two studies from Southern China in general hospital patients with 21/183 patients (1994–1997) and 13/257 patients (reported in 2006) with CDI (no diagnostic criteria reported). Another study from Beijing identified 36 cases among 71,428 in-patients from 1998 to 2001 [35]. Further studies were limited to special groups of patients with malignancy and those receiving chemotherapy or stem cell transplantation, with incidence rates varying from 1.6% to 3.5%, with an exceptionally high rate (27%) reported in the stem cell transplant patient study [35]. In most studies a sampling frame was not reported which, in view of the low rate of testing in China, makes assessment of the true incidence of CDI impossible [35].

**Figure 1:**
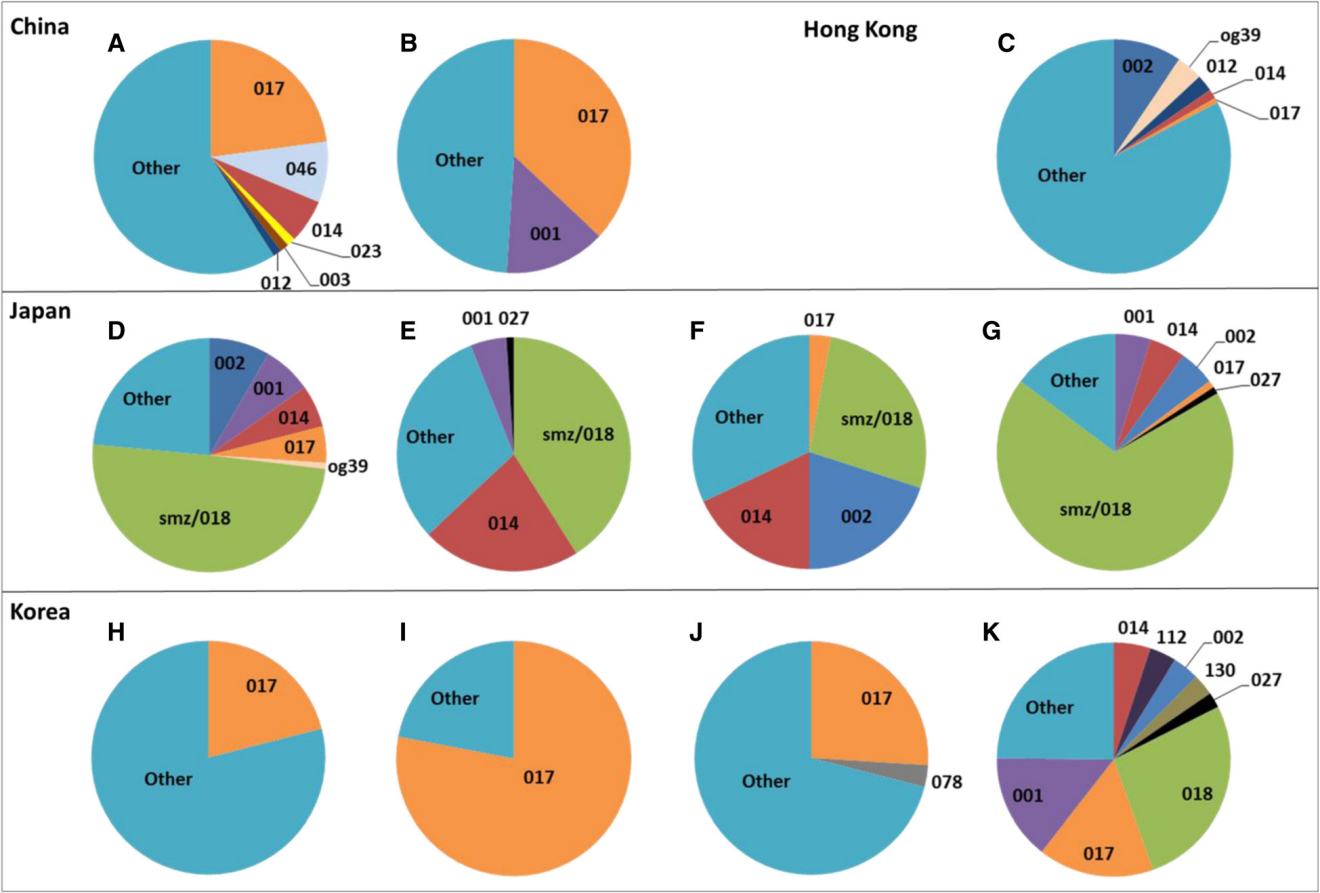
**Ribotype distributions for studies in China (A,B), Hong Kong (C), Japan (D-G) and Korea (H-K). A**: n = 75 isolates collected in 2008; 33 ribotypes identified [37]. **B**: n = 110, December 2008-May 2009; 16 ribotypes [39]. **C**: n = 345, 2009; 106 ribotypes [40]. **D**: n = 87, March 1996-November 1999; 12 ribotypes [8]. **E**: n = 148, November 1999-October 2004; 26 ribotypes [16]. **F**: n = 71, April 2005-March 2008; 20 ribotypes [14]. **G**: n = 87, 2003–2007; 18 ribotypes [17]. **H**: n = 187, 1980–2006; 39 ribotypes [27]. **I**: n = 105, 1995–2008; 11 ribotypes [32]. **J**: n = 337 toxigenic isolates, 2006–2008; 50 ribotypes [31]. **K**: n = 194, 2009–2010; 54 ribotypes [19].

The most comprehensive Chinese study of CDI was conducted between March 2007 and April 2008 at a 1,216 bed hospital in Shanghai [36-38]. During the study period, 42,936 patients were discharged and 587 patients had stool samples submitted for testing by toxin assay and culture [36]. Overall the incidence of CDI was 17.1 per 10,000 admissions [37]. CDI was mild, possibly due to the younger mean age of patients (62.8 years) compared with a large European survey where 63% were ≥65 years [20]. Fifty-six isolates from this study were aggregated with further unspecified isolates to create a collection of 75 [38]. The most common ribotypes were 017, 012 and 046 (Figure 1).

In the absence of ribotyping studies other than that of Huang *et al.*, a recent report of MLST typing on 69 *C. difficile* strains mainly from Beijing provides some further insight on the molecular epidemiology of CDI in China [41]. The equivalent ribotypes of the most common sequence types (ST37, ST35, ST54) found were 017 (23%), 046 (23%) and 012 (17%), respectively [42]. The collection included 16 Guangzhou isolates from the 1980s comprised of the ribotype equivalents: 017 (9 isolates), 046 (1 isolate), 020 (5 isolates) and ST119 (1 isolate). This suggests that ribotypes 017, 046 and perhaps 012 are the most common in mainland China, a pattern that differs to other world regions.

#### Taiwan

A similar situation to Japan and China is found in Taiwan, where testing is rare so the true incidence of CDI cannot be defined. The number of CDI cases increased by 5–6 times in patients ≥65 years between 2003 and 2007 according to one study [43]. Increasing awareness and testing probably contributed to the dramatic rise, as another report by Chan *et al*. demonstrates. They found the proportion of positive cultures remained constant at ~10% while testing increased from 2002 to 2009 as awareness of CDI increased [44]. The incidence rate found at a northern Taiwan hospital was 0.45/1,000 patient days overall, and 7.9/1,000 patient days in ICU wards [45]. Ribotype 017 strains were found (6%) in a sample of 142 Taiwanese isolates where 57 ribotypes were identified [46], however other internationally recognised ribotypes of Taiwanese isolates have not been reported. While binary toxin-positive isolates were detected, ribotypes 027 and 078 have not been reported from Taiwan [47].

#### Hong Kong

CDI has been better recognised and studied for longer in Hong Kong than in mainland China and Taiwan. A survey at Queen Mary Hospital, Hong Kong in 1996–7 reported 100/3,112 patients with diarrhoea positive by culture for *C. difficile*[48]. A more recent survey at the same hospital using a tissue culture cytotoxin assay between September and December 2008 detected 37/723 positive samples [49]. The number of patients diagnosed in 2003 was 82 compared to 66 in 2008, suggesting the overall number of cases was constant. Testing for CDI increased following the isolation of ribotype 027 among one of 12 hyper-toxigenic (detectable cytotoxin at 100-fold dilution) isolates identified in a 3-month study in a university hospital in 2008 [49].

There are little data on ribotyping in Hong Kong, the most comprehensive being a retrospective study of 345 isolates from 307 patients in 2009 from a healthcare region spanning five hospitals. Unusually, 70% were of a pattern not represented by 23 of the most internationally common ribotypes, with a further 11.6% being non-typable (Figure 1) [40]. Ribotype 002 represented 9.4% of strains, presumed to be causing cross-infection as the incidence between 2004 and 2008 was 0.53/1,000 admissions rising in 2009 to 0.95/1,000 admissions. An elevated frequency of sporulation (20.2%) was found in 35 of these ribotype 002 isolates compared to 3.7% for 56 randomly selected isolates of other ribotypes [40]. Ribotype 017 strains were also found at a prevalence of 0.7% [40]. Ribotype 027 was first detected in 2008 in stool of a patient on steroids with no history of travel in the previous 1.5 years and, as yet, there have been no further reports of 027 from Hong Kong [49].

### Southeast Asia

#### Philippines

The underestimation of the role of *C. difficile* in enteric disease in Asia was demonstrated recently by a study in the Philippines. Historically, patients with colitis were diagnosed with amoebic colitis, presumed to be caused by *Entamoeba histolytica*. However, 43.6% of colitis cases were positive for *C. difficile* when testing by enzyme immunoassay (EIA) for toxin A/B was introduced [50]. Given that metronidazole is the standard treatment for both CDI and amoebic colitis, CDI would be masked if testing was not carried out.

#### Thailand

In the earliest report of CDI in Thailand in 1990, faecal cytotoxin was detected in 52.5% of 206 diarrhoeal patients while culture gave low recovery rates (4.8%) over a 26-month period. Cytotoxin was detected in stool of 61% of antibiotic-treated patients, and 51% of non-antibiotic-treated patients [51]. In contrast, Thamlikitkul *et al*. reported low detection of toxin A by EIA in clindamycin-treated (10/140 patients) and patients treated with beta-lactams (10/140) [52]. The difference in prevalence found in both studies was probably due to the use of different toxin assays.

The prevalence of *C. difficile* in antibiotic-associated diarrhoea (AAD) patients was reported as 18.64% by culture and 44-46% by PCR for *tcdA* and *tcdB* on the same stool samples, showing the need for both culture and testing for presence of toxin [53]. Similarly, a recent study in Bangkok found that combining toxin EIAs with direct *tcdB* PCR on stool increased positive diagnoses two-fold, compared with toxin EIAs alone [54].

Wongwanich *et al*. found a greater prevalence of CDI in 201 HIV-positive patients (58.8%) than in 271 HIV-negative patients (36.5%), and six PFGE subtypes were found [55]. Again, toxin A EIA alone gave lower prevalence than when combined with culture. Another study on AIDS patients where only toxin A EIA was performed reported a lower prevalence of CDI (16/102 patients) [56]. The discrepancies between studies where lower prevalence is reported when a toxin A assay alone was used suggest it is likely that toxin A-negative strains were widespread.

Wongwanich *et al.* reported on a collection of 77 *C. difficile* isolates from 44 asymptomatic children and infants, and 33 diarrhoeal adult patients. In this study, *tcdA* PCR-negative isolates predominated, numbering 57 overall and 18/33 adult diarrhoeal isolates. Fourteen PFGE pulsotypes and eight subtypes were found [57]. No data on circulating ribotypes have been reported.

#### Malaysia

Reports of CDI in Malaysia are rare. In the most informative study in Malaysia to date, toxin A/B assays on 175 stool samples from inpatients with AAD were performed in a tertiary hospital in north-eastern Malaysia. Twenty-four (13.7%) were positive for toxin, with the majority of infected patients aged >50 years [58]. No ribotyping or other molecular analysis has been reported on Malaysian *C. difficile* isolates.

#### Indonesia

Like Malaysia, reports on CDI in Indonesia are uncommon. An aetiology study of diarrhoea in Indonesian children identified *C. difficile* in 1.3% of stool samples tested. Toxin A EIA only was performed so the true prevalence of *C. difficile* may have been greater [59]. The only molecular study included eight isolates from Indonesia, five of which were toxinotype VIII and ribotype 017, and grouped with international 017 epidemic strains. Two were A^+^B^+^ toxinotype 0, and one A^-^B^+^ isolate was binary toxin-positive, toxinotype XVI [15].

#### Singapore

A 50-month aetiological study (1985 to 1989) of diarrhoea in 4,508 patients at the National University Hospital found *C. difficile* in 35 of only 365 cases where *C. difficile* culture was requested. The incidence rate of CDI in Singapore General Hospital was 3.2/1,000 admissions, more common in males and patients >50 years. Culture of stool samples identified cases (43%) which were negative by toxin A/B EIA. CDI was three times more common in Malay patients than in patients of Indian heritage [60]. An increase in incidence of CDI was observed in a 1,200 bed general hospital from 2001 to 2006. The incidence rate rose from 1.49 cases per 10,000 patient days to 6.64 per 10,000 patient days, with a concurrent increase in positive toxin assays on stool [61]. A broader surveillance study in three hospitals found that CDI incidence decreased from 0.52/1,000 patient days in 2006 to 0.3/1,000 patient days in 2008, as testing increased over the same time period [62]. Binary toxin-positive strains have been reported, including 027 strains, which have caused sporadic hospital-acquired disease [63].

### South Asia

#### India

An early report found 21/93 AAD cases were positive for *C. difficile* by culture and toxin assay in Nehru Hospital in 1983–1984 [64]. In Calcutta, *C. difficile* was isolated in 38/341 hospitalised patients with acute diarrhoea over 1 year [65]. A hospital in Delhi reported CDI in 26/156 diarrhoeal hospitalised patients, detected by culture and toxin A EIA [66]. Infection control measures subsequently introduced at this hospital apparently reduced incidence of CDI by more than 50% over 5 years [67]. A retrospective review by Ingle *et al*. in a Mumbai hospital found 17/99 patients between 2006 and 2008 were diagnosed with CDI by toxin A/B EIA [68]. The most recent report found a prevalence of non-toxigenic *C. difficile* of 12.6% among 79 hospitalised patients, five of whom subsequently developed diarrhoea with positive culture and toxin assay. The study group also detected widespread contamination of surfaces on beds (51%) and hands of hospital workers (62.5%) [69]. Several reports exist of acute diarrhoea in hospitalised children (7-11%) caused by *C. difficile*[70-72]. The molecular epidemiology of *C. difficile* strains in India is not currently known.

#### Bangladesh

In the 1990s, an aetiological study found that 13/814 children admitted to hospital with diarrhoea were infected with *C. difficile* (diagnosed by cell cytotoxin assay). Seven of the cases were concurrently infected with another diarrhoeal agent [73]. Recent reports are not available for Bangladesh.

## Epidemiology

### Prevalent *C. difficile* ribotypes in Asia

Ribotyping data with internationally recognised nomenclature are available for China, Japan, Singapore, Hong Kong, Taiwan, and Korea. Overall, the most prevalent ribotypes in Asia appear to be 017, 018, 014, 002, and 001. While ribotypes 027 and 078 have caused outbreaks in North America and Europe, they are reported only to have caused sporadic cases of CDI in Asia so far, in Singapore, Hong Kong, Korea, and Japan [16,34,40,46,49,63,74]. Ribotype 078 has only been reported from Korea and China to date [31,37]. Another binary toxin-positive strain, ribotype 130, was recently reported from Korea [19].

Meanwhile, ribotype 017, A^-^B^+^, toxinotype VIII strains are widespread in Asia, and have caused epidemics worldwide (Figure 1). In China and Korea 017 is the most common ribotype in circulation, and is prevalent in Japan, Taiwan, and Hong Kong also [31,32,36,39]. Exposure to antineoplastic agents, use of nasal feeding tubes, and care in a particular hospital ward were associated with infection with 017 strains in one hospital in Japan [11]. Ribotype 017 strains have persisted in China and Taiwan while they appear to have declined in Korea (Figure 1).

In Japan, smz/018 appears to have persisted as the most common ribotype for over a decade (Figure 1). Ribotype smz/018 was the most prevalent strain isolated in a tertiary hospital in Seoul between September 2008 and January 2010 [33], indicating spread from Japan to Korea. Ribotype 018 caused outbreaks of CDI in Italy in 2007/2008 and is the fourth most prevalent ribotype in Europe at present [20,21]. It is not clear whether smz/018 is prevalent in other Asian countries, as comparative typing with a reference smz or 018 strain may not have been performed.

Ribotypes 017 and 018 have caused widespread disease in Asia and across the world. Unlike the other major epidemic strains 027 and 078, they do not produce binary toxin, and ribotype 018 does not appear to possess variant toxin genes [21]. Some other virulence factors may contribute to their spread. The resistance of ribotype 018 isolates to clindamycin and fluoroquinolones could contribute to their enhanced virulence [8,33,40]. Another virulence factor is high sporulation rate. The epidemic ribotype 002 isolates in Hong Kong sporulated at a higher rate than other isolates, allowing them to persist in the hospital environment and cause outbreaks of disease [40].

### Prevention and control

Two reports of infection control in Asian hospitals were found. A hospital in India introduced control measures including disinfection of surfaces, rapid detection of *C. difficile* by toxin assays, isolation of patients, controls on prescription of antibiotics and education of staff members. The incidence of CDI (initially 15% among cases of nosocomial diarrhoea) was reduced by 50% while the number of tests requested increased as health workers became more aware of CDI [67]. A hospital-wide computerised antimicrobial stewardship scheme was introduced in a hospital in Taiwan. While the incidence of some antibiotic resistant organisms decreased, the isolation rate of *C. difficile* remained constant at 10% [44], indicating that other infection control measures besides antimicrobial stewardship would be required to control CDI in hospitals.

Asia is going through a period of rapid demographic change. With its dense, growing population, infection control is a pertinent issue. As *C. difficile* now causes the majority of nosocomial disease in North America and Europe, control measures could be applied in Asia to prevent the same situation there. A number of issues exist which could contribute to the spread of CDI in Asia.

As wealth and the aged population are increasing, more people have access to hospital care and enter aged care facilities. It is likely that CDI incidence could increase as these high-risk populations increase in size. For example, modelling of the future age structure of the Chinese population suggests that there will be a larger population at risk for CDI. Using census data from 2005 (population 1.3 billion) when only 100 million individuals were ≥65 years old, by 2026 there will be 200 million individuals ≥65 years [75].

Antibiotic use in most Asian countries is poorly regulated. A review of Southeast Asian countries found that 47% of pneumonia cases do not receive an appropriate antibiotic, 54% of diarrhoea cases are unnecessarily treated with antibiotics, and 40% of antibiotics are prescribed in under-dose [76]. In many cases inappropriate antibiotics are prescribed without any laboratory test. Studies in India have found the most commonly prescribed antibiotics for cough and respiratory disease are fluoroquinolones, a known risk factor for CDI [76]. In addition, antibiotics are freely available without prescription in most Asian countries, leading to misuse in the community.

Given the free use of antibiotics by the general public it would be plausible that CA-CDI could be common in Asia. Studies in Asian countries have neglected to address the issue of CA-CDI, apart from two studies in Korea which found conflicting proportions of 7% and 59% of all CDI surveyed being community-acquired. It would be appropriate to monitor CA-CDI more closely in Asia in the future.

Despite widespread antibiotic use few studies in Asia have measured antimicrobial susceptibility of clinical *C. difficile* isolates. High resistance rates to moxifloxacin, and clindamycin have been found in isolates from Korea, Japan, Northern Taiwan and China (Table 1). Heteroresistance to metronidazole has been reported from China, warranting close monitoring (Table 1).

**Table 1:** **Antimicrobial resistance rates and MIC values for *Clostridium difficile *isolates from different countries**

**(Reference)**	**Number of isolates**	**[Resistance rate] (%), MIC_50_ (mg/L), MIC_90_ (mg/L)**							
		**Erythromycin**	**Clindamycin**	**Tetracycline**	**Moxifloxacin**	**Ciprofloxacin**	**Piperacillin/ tazobactam**	**Metronidazole**	**Vancomycin**
**China**									
[37]	75	[76], 128, 128	[66.7], 128, 128	[41.3], 4, 64	[45.3], 4, 128	[100], 64, 128	[0], <16/4, 16/4	[0], 0.25, 0.25	[0], 1, 2
[39]	110	[85.3], 128, 128	[88.1], 128, 128	[62.7], 16, 32	[61.8], 16, 128	[100], 64, 128	[0], 8/4, 16/4	[0]^a^, 0.125, 0.25	[0], 0.5, 1
**Hong Kong**									
[40]	35					[100]		0.5, 0.75	0.75, 1.5
**Japan**									
[77]	73	[87.7], >256, >256	[87.7], >256, >256			[100], >32, >32		[0], 0.19, 0.25	[0], 2, 4
[16]	72				[12]^b^	[100]		[0]	[0]
**Korea**									
[31]	120		[50], 128, >128		[42], 2, 16		[0], 8, 16	[0], 1,4	[0], 0.5, 1
[19]	111		[82]		[83]				
[33]	131		[67.9]		[82]			[0]	[0]
[78]	123		[75]		[85]				
**Singapore**									
[60]	68		[63], 8, >512					[0], 0.5, 1	[0], 1, 1
**Taiwan**									
[79]	60		[73.3], 16, 64	[41.7], 8, >16	[30]	[100]			
[47]	113		[46], 4, >256		[16]			[0]	

^a^ 18 isolates showed heteroresistance to metronidazole, ^b^subset of 25 isolates tested

Production and consumption of meat products is also increasing in Asia [80]. Intensive farming of poultry, seafood and swine is already in place, and increasing with worldwide demand [81]. The risk of *C. difficile* colonisation and/or infection in animals would most likely increase with intensive farming practices including crowding of animals and prophylactic antibiotic use. Thus contamination of food products and animal-human transmission could occur. To date, no reports have been made of *C. difficile* in the environment or animals, apart from five cases of fulminant colitis caused by ribotype 078 in thoroughbred racehorses in Japan [82], and the isolation of *C. difficile* from 2/ 250 (0.8%) swine faecal samples from 25 pig farms, also in Japan [83].

## Review

According to the existing evidence, CDI occurs at similar rates in Asia as in other continents where CDI is more commonly recognised and researched. The molecular epidemiology of *C. difficile* strains in Asia indicates a persisting predominance of variant A^-^B^+^ ribotype 017 strains and ribotype 018 strains (Figure 1). Binary toxin-positive strains have rarely been isolated to date despite the proliferation of ribotypes 078 and 027 in Europe and North America. Favouring toxin A EIAs for diagnostic methods is not optimal for the Asian region due to the predominance of A^-^B^+^ strains. Broader surveillance monitoring CA-CDI and *C. difficile* in animals will enhance our understanding of the epidemiology of CDI in the region.

## Conclusions

CDI is not widely recognised in Asia so in consequence the extent of the disease is not known. Although relatively few studies on *C. difficile* have been performed in Asia, what work has been done demonstrates that CDI is a significant cause of nosocomial disease in Asian countries. It appears that awareness is increasing and testing and surveillance are on the rise. Routine testing is required to inform on the prevalence of CDI throughout the region. The widespread prevalence of the 017 group of A^-^B^+^ strains in Asian countries shows that assays for toxin B or the *tcdB* genes are preferable to toxin A assays for diagnosis of CDI. The more virulent epidemic strains 027 and 078 do not appear to have become established in Asia, while ribotype 017 and smz/018 strains have caused epidemics.

Widespread unregulated antibiotic use and inappropriate prescribing in SE Asian countries indicates that CDI could be widespread in those regions where surveillance is currently lacking. Asia may be facing a “perfect storm” as heavy usage of antibiotics combines with an ageing increasingly hospitalised population. Increasing laboratory capacity in the region as well as improving surveillance should be seen as essential in preventing unnecessary morbidity and mortality.

## Abbreviations

CDI: *Clostridium difficile* infection; PMC: Pseudomembranous colitis; PFGE: Pulsed-field gel electrophoresis; PCR: Polymerase chain reaction; PPI: Proton pump inhibitor; CA-CDI: Community-acquired *Clostridium difficile* infection; EIA: Enzyme immunoassay; AAD: Antibiotic-associated diarrhoea.

## Competing interests

The authors declare that they have no competing interest.

## Authors’ contributions

DAC performed literature searches, analysed and interpreted data and drafted the manuscript. PMH planned and designed the review, interpreted data, drafted and revised the manuscript. TVR planned and designed the review and revised the manuscript. All authors read and approved the final manuscript.
